# Corosolic Acid Induces Non-Apoptotic Cell Death through Generation of Lipid Reactive Oxygen Species Production in Human Renal Carcinoma Caki Cells

**DOI:** 10.3390/ijms19051309

**Published:** 2018-04-27

**Authors:** Seon Min Woo, Seung Un Seo, Kyoung-jin Min, Seung-Soon Im, Ju-Ock Nam, Jong-Soo Chang, Shin Kim, Jong-Wook Park, Taeg Kyu Kwon

**Affiliations:** 1 Department of Immunology, School of Medicine, Keimyung University, Daegu 42601, Korea; woosm724@gmail.com (S.M.W.); ssu3885@gmail.com (S.U.S.); Kyoungjin.min@gmail.com (K.-j.M.); god98005@dsmc.or.kr (S.K.); j303nih@dsmc.or.kr (J.-W.P.); 2 Physiology of Department, School of Medicine, Keimyung University, Daegu 42601, Korea; ssim73@kmu.ac.kr; 3 Department of Food Science and Biotechnology, Kyungpook National University, Daegu 41566, Korea; namjo73@gmail.com; 4 Department of Life Science, College of Science and Technology, Daejin University, Kyeonggido 11159, Korea; jchang@daejin.ac.kr

**Keywords:** corosolic acid, non-apoptotic cell death, α-tocopherol, lipid ROS

## Abstract

Corosolic acid is one of the pentacyclic triterpenoids isolated from *Lagerstroemia speciose* and has been reported to exhibit anti-cancer and anti-proliferative activities in various cancer cells. In the present study, we investigated the molecular mechanisms of corosolic acid in cancer cell death. Corosolic acid induces a decrease of cell viability and an increase of cell cytotoxicity in human renal carcinoma Caki cells. Corosolic acid-induced cell death is not inhibited by apoptosis inhibitor (z-VAD-fmk, a pan-caspase inhibitor), necroptosis inhibitor (necrostatin-1), or ferroptosis inhibitors (ferrostatin-1 and deferoxamine (DFO)). Furthermore, corosolic acid significantly induces reactive oxygen species (ROS) levels, but antioxidants (*N*-acetyl-l-cysteine (NAC) and trolox) do not inhibit corosolic acid-induced cell death. Interestingly, corosolic acid induces lipid oxidation, and α-tocopherol markedly prevents corosolic acid-induced lipid peroxidation and cell death. Anti-chemotherapeutic effects of α-tocopherol are dependent on inhibition of lipid oxidation rather than inhibition of ROS production. In addition, corosolic acid induces non-apoptotic cell death in other renal cancer (ACHN and A498), breast cancer (MDA-MB231), and hepatocellular carcinoma (SK-Hep1 and Huh7) cells, and α-tocopherol markedly inhibits corosolic acid-induced cell death. Therefore, our results suggest that corosolic acid induces non-apoptotic cell death in cancer cells through the increase of lipid peroxidation.

## 1. Introduction

Reactive oxygen species (ROS) are formed as a byproduct of the metabolism of oxygen and have critical roles in cell homeostasis and signaling [1]. A reasonable amount of ROS can promote cancer cell proliferation, whereas generation of extreme ROS can cause oxidative stress, leading to induction of cancer cell death [2]. Therefore, regulation of ROS homeostasis is important for cancer cell growth and survival [3]. ROS can attack lipids and induce lipid peroxidation [4]. Lipid peroxidation induces oxidative degradation of lipids, eventually resulting in damage of cancer cells [5]. It is involved in the underlying mechanisms of several diseases, such as cancer, senescence, and neurodegenerative diseases [6,7].

Corosolic acid is a pentacyclic triterpenoid discovered in traditional Asian medical herbs such as *Lagerstroemia speciose* [8,9]. Corosolic acid has been investigated for its anti-diabetic activity in animal models through development of glucose metabolism [10]. Moreover, it has been reported for anti-tumor and anti-proliferative activities against many human cancer cells. For examples, corosolic acid induces loss of mitochondrial membrane potential and caspase activation in cervix adenocarcinoma [11], colon cancer [12], leukemia [13], and osteosarcoma cells [14]. In addition, corosolic acid increases intracellular ROS production, leading to induction of apoptosis in lung adenocarcinoma cells [15]. In human gastric cancer cells, corosolic acid induces cell cycle arrest through down-regulation of human epidermal growth factor receptor 2 (HER2) signaling and increases apoptosis [16]. Moreover, corosolic acid inhibits cell proliferation in glioblastoma cells via suppression of signal transducer and activator of transcription 3 (STAT3) signaling [17]. However, the anti-cancer activity of corosolic acid in human renal carcinoma cells has not yet been investigated.

In this study, we investigated whether corosolic acid induces cell death, and identified the molecular mechanism of corosolic acid-induced cell death in human renal carcinoma Caki cells.

## 2. Results

### 2.1. Corosolic Acid Induces Caspase-Independent Cell Death in Renal Carcinoma Caki Cells

**Figure 1:**
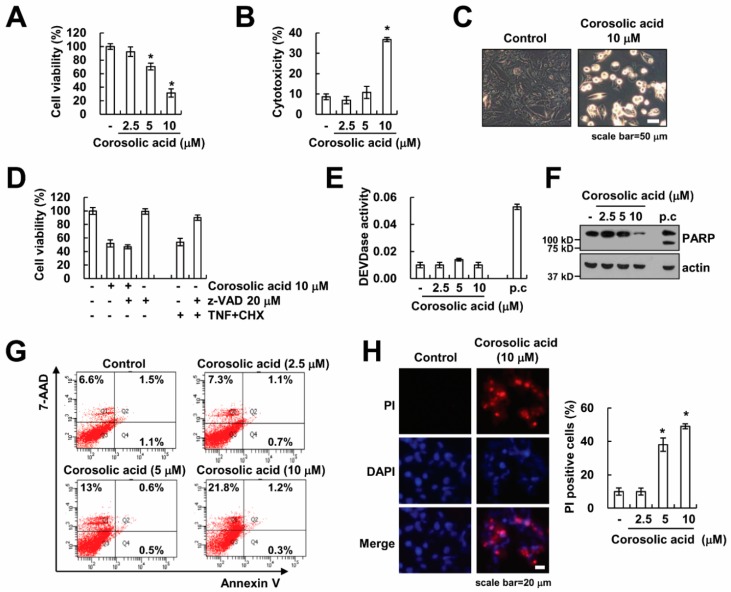
Corosolic acid induces non-apoptotic cell death through caspase-independent manner. (**A**,**B**) Caki cells were treated with 2.5, 5, or 10 µM corosolic acid for 24 h. 2,3-Bis(2-methoxy-4-nitro-5-sulfophenyl)-2H-tetrazolium-5-carboxanilide (XTT) assay was used to detect the cell viability (**A**); Lactate dehydrogenase (LDH) release assay was used to detect the cell cytotoxicity (**B**); (**C**) Caki cells were treated with 10 µM corosolic acid for 24 h. We detected the cell morphology using interference light microscopy; (**D**) Caki cells were treated with 10 µM corosolic acid or 10 ng/mL TNF-α plus 5 µg/mL cycloheximide (CHX) for 24 h in the presence or absence of 20 µM z-VAD-fmk (z-VAD). XTT assay was used to detect the cell viability; (**E**–**G**) Caki cells were treated with 2.5, 5, or 10 µM corosolic acid for 24 h (p.c: positive control; 10 ng/mL TNF-α plus 5 µg/mL CHX for 24 h). Caspase activities were detected using a kit, as described in material and methods (**E**); Western blotting was used to detect the protein levels of PARP and actin (**F**); Flow cytometry was used to detect the Annexin V/7-AAD staining (**G**); (**H**) Caki cells were treated with 10 µM corosolic acid for 24 h. After treatment with corosolic acid, cells were stained with propidium iodide (PI) and 4′,6-diamidino-2-phenylindole (DAPI), and fluorescence microscope (left panel) or flow cytometry (right panel) was used to detect PI uptake. The values in the graphs (**A**,**B**,**D**,**E**,**H**) represent the mean ± SD of three independent samples. * *p* < 0.01 compared to the control.

Because corosolic acid has an anti-cancer effect in various cancer cells [11,12,13,15,16,18], we examined whether corosolic acid induces cell death in renal carcinoma Caki cells. Corosolic acid decreased cell viability and increased cell cytotoxicity in a dose-dependent manner (Figure 1A,B). Moreover, corosolic acid increased morphologically dying cells (Figure 1C). Next, we investigated whether activation of caspases was associated with corosolic acid-induced cell death. Pretreatment with z-VAD-fmk (z-VAD), the pan-caspase inhibitor, inhibited cell death induced by TNF-α, with cycloheximide (CHX) as a positive control [19]. However, treatment of z-VAD had no effect on corosolic acid-induced cytotoxicity (Figure 1D). Furthermore, corosolic acid did not induce activation of caspase-3, whereas TNF-α plus CHX increased caspase-3 activity (Figure 1E). To confirm caspase independent cell death by corosolic acid, we checked the hallmarks of apoptosis, such as cleavage of poly (ADP-ribose) polymerase (PARP). As shown in Figure 1F, corosolic acid did not increase PARP cleavage. To identify apoptotic and necrotic cells, cells were stained with Annexin V/7-Aminoactinomycin D (7-AAD) and propidium iodide (PI) [20]. Annexin V fluorescence can detect apoptotic cells, while 7-AAD fluorescence can detect necrotic cells. Corosolic acid induced a 7-AAD-positive population (Figure 1G). Moreover, uptake of PI also increased in corosolic acid-treated cells (Figure 1H). Therefore, these results indicate that corosolic acid induces caspase-independent non-apoptotic cell death.

### 2.2. Corosolic Acid-Induced Cell Death Is not Associated with Necroptosis

**Figure 2:**
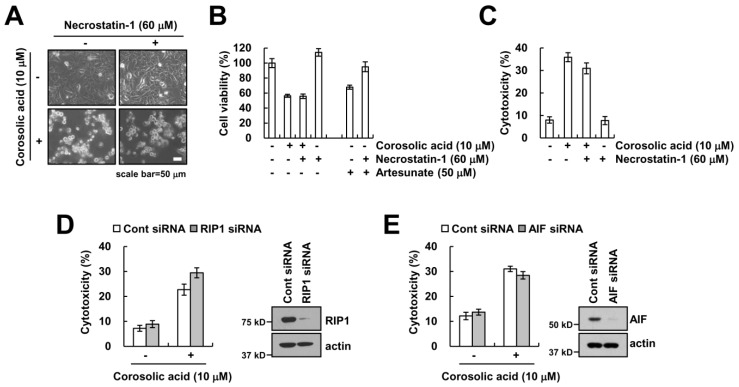
Corosolic acid-induced cell death is independent of necroptosis. (**A**–**C**) Caki cells were treated with 10 µM corosolic acid or 50 µM artesunate (positive control) in the presence or absence of 60 µM necrostatin-1. We detected the cell morphology using interference light microscopy (**A**); XTT assay was used to detect the cell viability (**B**); LDH release assay was used to detect the cell cytotoxicity (**C**); (**D**,**E**) Caki cells were transiently transfected with siRNA against control, RIP1, and AIF. After 24 h, cells were treated with 10 µM corosolic acid for 24 h. LDH release was used to detect the cell cytotoxicity, and western blotting was used to detect the protein levels of RIP1, AIF, and/or actin. The values in the graphs (**B**,**C**,**D**,**E**) represent the mean ± SD of three independent samples.

To further confirm whether corosolic acid-induced cell death is involved in necrotic cell death, we used necrostatin-1, a selective inhibitor of necroptosis. Necrostatin-1 did not affect corosolic acid-mediated reduction of cell viability and induction of cell cytotoxicity (Figure 2A–C). Receptor-interacting protein kinase1 (RIP1) plays critical role in induction of necroptosis [21]. Knock-down of RIP1 expression by siRNA did not inhibit cytotoxicity in corosolic acid-treated cells (Figure 2D). Previous studies reported that apoptosis-inducing factor (AIF) is a key regulator of caspase-independent necroptosis [22,23]. However, we found that knock-down of AIF expression using siRNA had no effect on the increase of cytotoxicity by corosolic acid (Figure 2E). Therefore, these results suggest that RIP1-and AIF-mediated necroptosis are not involved in corosolic acid-mediated cell death.

### 2.3. The Generation of ROS Is not Involved in Corosolic Acid-Induced Cell Death

**Figure 3:**
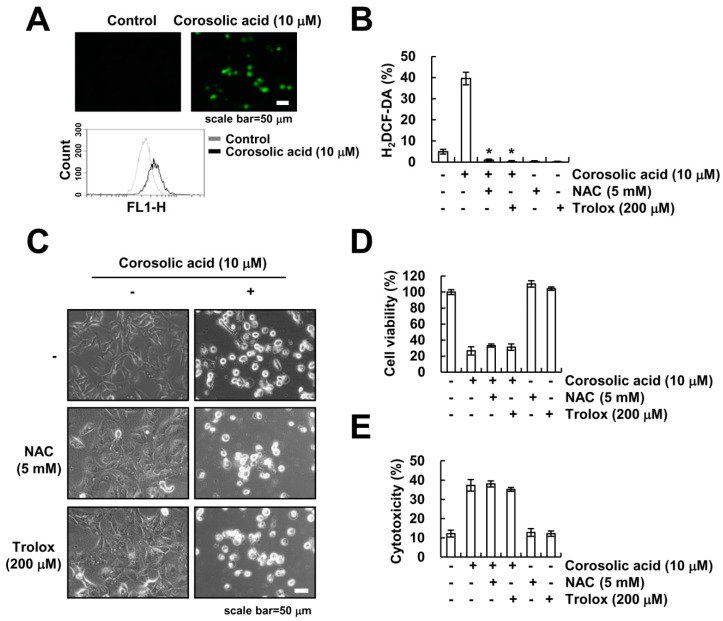
The generation of ROS is not involved in the induction of corosolic acid-induced non-apoptotic cell death. (**A**) Caki cells were treated with 10 µM corosolic acid for 9 h. After treatment with corosolic acid, cells were loaded with H_2_DCF-DA fluorescent dye, and fluorescence microscope (upper panel) or flow cytometry (lower panel) was used to detect intracellular ROS levels; (**B**–**E**) Caki cells were pre-treated with 5 mM NAC and 200 µM trolox for 30 min and were then treated with 10 µM corosolic acid for 9 h (**B**) and 24 h (**C**–**E**); Cells were loaded with H_2_DCF-DA fluorescent dye, and flow cytometry was used to detect intracellular ROS levels (**B**); We detected the cell morphology using interference light microscopy (**C**); XTT assay was used to detect the cell viability (**D**), and LDH release assay was used to detect the cell cytotoxicity (**E**); The values in the graphs (**B**,**D**,**E**) represent the mean ± SD of three independent samples. * *p* < 0.01 compared to corosolic acid-treated cells.

The generation of ROS plays a critical role in many types of cancer cell death [24,25,26]. A previous study reported that corosolic acid increases intracellular ROS in human lung adenocarcinoma cells [15]. Therefore, we investigated whether ROS signaling is involved in corosolic acid-induced cell death. Corosolic acid markedly increased generation of ROS (Figure 3A). Moreover, pretreatment with ROS scavenger (*N*-acetyl-l-cysteine (NAC) and trolox) significantly inhibited corosolic acid-induced ROS production (Figure 3B). However, NAC and trolox had no effect on corosolic acid-induced cell death (Figure 3C–E). Therefore, these results indicate that ROS signaling is not associated with corosolic acid-induced cell death.

### 2.4. Corosolic Acid-Mediated Cell Death Is Independent of Ferroptosis

**Figure 4:**
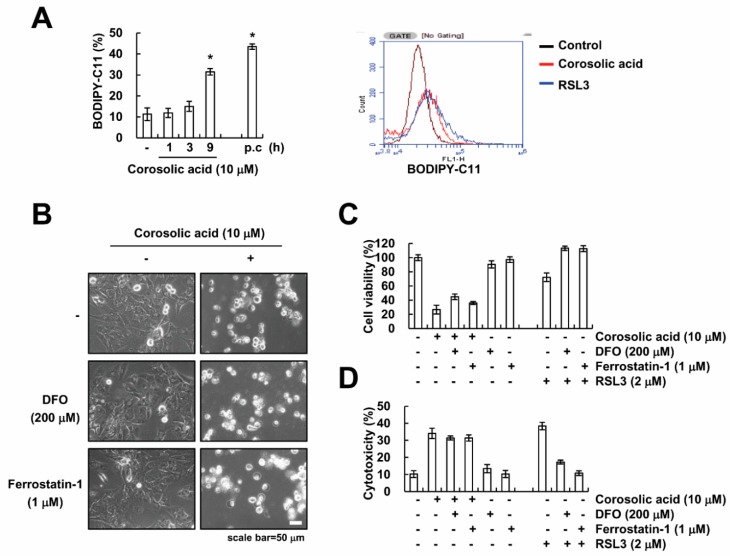
Corosolic acid-induced cell death is not associated with induction of ferroptosis. (**A**) Caki cells were treated with 10 µM corosolic acid for indicated time periods (left panel) or 9 h (right panel) (p.c: positive control; 2 µM RAS-selective lethal 3 (RSL3) for 9 h). After treatment with corosolic acid, cells were loaded with BODIPY-C11 fluorescent dye, and flow cytometry was used to detect lipid peroxidation; (**B**–**D**) Caki cells were pre-treated with 200 µM DFO and 1 µM ferrostatin-1 for 30 min and were then treated with 10 µM corosolic acid or 2 µM RSL3 for 24 h. We detected the cell morphology using interference light microscopy (**B**); XTT assay was used to detect the cell viability (**C**), and LDH release assay was used to detect the cell cytotoxicity (**D**); The values in the graphs (**A**,**C**,**D**) represent the mean ± SD of three independent samples. * *p* < 0.01 compared to the control.

Ferroptosis is a newly identified form of cell death that results from iron-dependent lipid peroxidation [27]. First, we measured the induction of lipid peroxidation by flow cytometry using BODIPY-C11 fluorescence dye. Corosolic acid markedly increased lipid peroxidation (Figure 4A). Next, to examine whether corosolic acid induces ferroptosis, we used deferoxamine (DFO) as an iron chelator, and ferrostatin-1 as a specific inhibitor of ferroptosis [27,28]. However, pretreatment with DFO and ferrostatin-1 did not reverse corosolic acid-induced decrease of cell viability and increase of cytotoxicity (Figure 4B–D). These data suggest that corosolic acid-induced cell death is not associated with iron-dependent ferroptosis.

### 2.5. α-Tocopherol Protects Against Corosolic Acid-Induced ROS Generation, Lipid Peroxidation, and Cell Death

**Figure 5:**
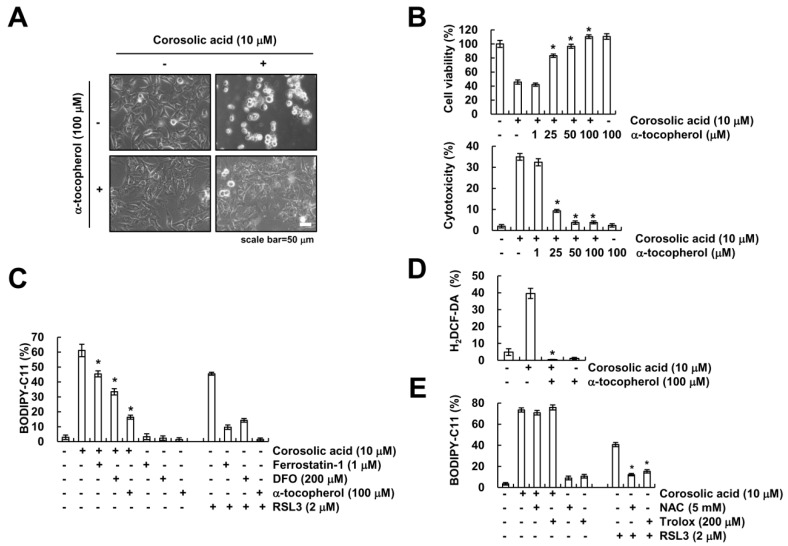
α-tocopherol inhibits corosolic acid-induced lipid peroxidation and cell death. (**A**,**B**) Caki cells were pre-treated with the indicated concentrations of α-tocopherol for 30 min and were then treated with 10 µM corosolic acid for 24 h. We detected the cell morphology using interference light microscopy (**A**); XTT assay and LDH release assay were used to detect the cell viability and cell cytotoxicity, respectively (**B**); (**C**) Caki cells were pre-treated with 200 µM DFO, 1 µM ferrostatin-1, and 100 µM α-tocopherol for 30 min and were then treated with 10 µM corosolic acid or 2 µM RSL3 for 9 h. After treatment with corosolic acid, cells were loaded with BODIPY-C11 fluorescent dye, and flow cytometry was used to detect lipid peroxidation; (**D**) Caki cells were pre-treated with 100 µM α-tocopherol for 30 min and were then treated with 10 µM corosolic acid for 9 h. After treatment with corosolic acid, cells were loaded with H_2_DCF-DA fluorescent dye, and flow cytometry was used to detect intracellular ROS levels; (**E**) Caki cells were pre-treated with 5 mM NAC and 200 µM trolox for 30 min and were then treated with 10 µM corosolic acid or 2 µM RSL3 for 9 h. After treatment, cells were loaded with BODIPY-C11 fluorescent dye, and flow cytometry was used to detect lipid peroxidation. The values in the graphs (**B**,**C**,**D**,**E**) represent the mean ± SD of three independent samples. * *p* < 0.01 compared to corosolic acid-treated cells.

Previous studies reported that α-tocopherol, a lipophilic antioxidant, reduces ferroptosis inducer-mediated lipid peroxidation [29,30]. To this end, we investigated the effect of α-tocopherol on corosolic acid-induced cell death. Interestingly, α-tocopherol markedly protected corosolic acid-mediated cell death (Figure 5A,B). To confirm the involvement of lipid peroxidation on cell death by corosolic acid, we checked lipid peroxidation. α-Tocopherol markedly inhibited increase of lipid peroxidation by corosolic acid (Figure 5C), but Ferrostatin-1 and DFO partially inhibited corosolic acid-mediated lipid peroxidation (Figure 5C). Moreover, corosolic acid-induced intracellular ROS generation was inhibited by α-tocopherol treatment (Figure 5D). In addition, we found that NAC and trolox inhibited RSL3-induced lipid oxidation, but both antioxidants had no effect on corosolic acid-induced lipid oxidation (Figure 5E). Therefore, these data indicate that α-tocopherol reduces corosolic acid-induced lipid peroxidation, ROS generation, and cell death.

### 2.6. Effect of Corosolic Acid on Cell Death in Other Cancer and Normal Cells

**Figure 6:**
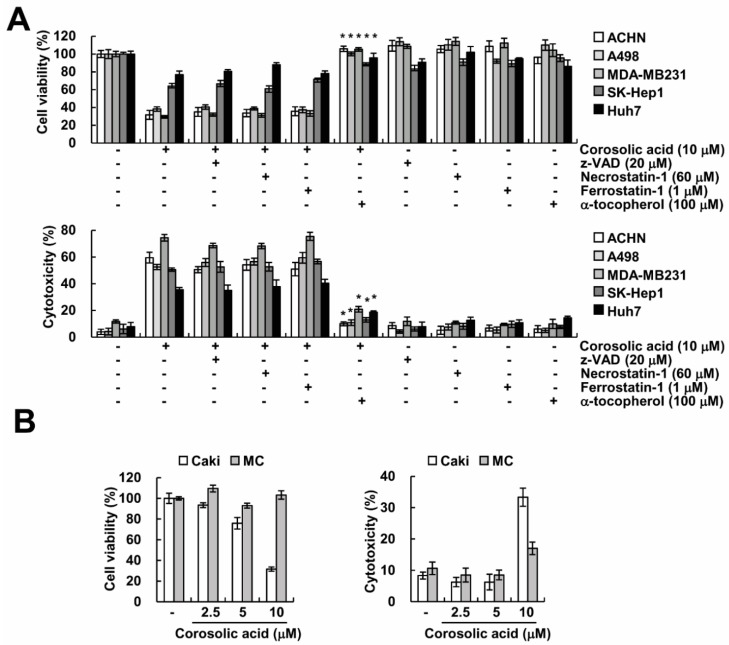
The effects of the corosolic acid-induced cell death in other carcinoma and normal cells. (**A**) Cancer cells were pre-treated with 20 µM z-VAD, 60 µM necrostatin-1, 1 µM ferrostatin-1, and 100 µM α-tocopherol for 30 min and were then treated with 10 µM corosolic acid for 24 h. XTT assay and LDH release assay were used to detect the cell viability (upper panel) and the cell cytotoxicity (lower panel); (**B**) Caki and mesangial cell (MC) cells were treated with 10 µM corosolic acid for 24 h. XTT assay and LDH release assay were used to detect the cell viability and the cell cytotoxicity; The values in the graphs (**A**,**B**) represent the mean ± SD of three independent samples. * *p* < 0.01 compared to corosolic acid-treated cells.

Further, we demonstrated whether corosolic acid enhances non-apoptotic cell death in other cancer cells. As shown in Figure 6A, corosolic acid induced suppression of cell viability and induction of cytotoxicity in renal carcinoma cells (ACHN and A498), breast cancer cells (MDA-MB231) and hepatocellular carcinoma cells (SK-Hep1 and Huh7). α-Tocopherol significantly inhibited corosolic acid-mediated cell death in various cancer cells (Figure 6A). However, other cell death inhibitors (z-VAD (apoptosis), necrostatin-1 (necroptosis), and ferrostatin-1 (ferroptosis)) did not affect corosolic acid-induced cell death (Figure 6A). In contrast, corosolic acid had no effect on cell viability and cytotoxicity in normal human mesangial cells (MC) (Figure 6B). Taken together, these data suggest that corosolic acid could induce non-apoptotic cell death in many cancer cells.

## 3. Discussion

In this study, we found that corosolic acid induces non-apoptotic cell death in cancer cells. In this event, lipid peroxidation was associated with corosolic acid-mediated cell death. Interestingly, corosolic acid-induced cell death was inhibited by α-tocopherol, but not apoptosis inhibitor (z-VAD), necroptosis inhibitor (necrostatin-1), or ferroptosis inhibitors (ferrostatin-1 and DFO). Our results suggested that corosolic acid induced lipid peroxidation-dependent non-apoptotic cell death.

Previous studies have reported that corosolic acid could induce caspase-dependent apoptosis through Bax activation and cytochrome *c* release in many cancer cells [11,12,13,14]. In our study, we found that corosolic acid induced cell death, but z-VAD did not inhibit cell death by corosolic acid in human renal (Caki, ACHN, and A498), breast (MDA-MB231), and hepatocellular (SK-Hep1 and Huh7) carcinoma cells (Figure 1D and Figure 6A). Moreover, corosolic acid did not affect caspase-3 activity and cleavage of PARP (Figure 1E,F). Therefore, our data indicate that corosolic acid induced caspase-independent non-apoptotic cell death. Although corosolic acid induced cell death in various cancer cells, the mode of corosolic acid-mediated cell death is dependent on cell contexts and cell types.

Programed cell death is classified by activation of caspases, including apoptosis and necroptosis. Necroptosis is a form of non-apoptotic cell death which is requires RIP1 and RIP3 activity, leading to formation of necrosome-dependent cell death [31,32]. We examined the effect of necrostatin-1 on corosolic acid-induced cell death. The pretreatment with necorostatin-1 did not block corosolic acid-induced-cell death (Figure 2A,C). Moreover, cell death induced by corosolic acid was not inhibited after knock-down of RIP1 by siRNA (Figure 2D). Therefore, our data suggest that corosolic acid-induced cell death is not associated with RIP1-dependent necroptosis. Ferroptosis is a newly identified type of cell death and results from iron-dependent lipid peroxidation [27]. As shown in Figure 4A, corosolic acid also increased lipid peroxidation in human renal carcinoma Caki cells. However, inhibitors of ferroptosis, DFO and ferrostatin-1, did not inhibit corosolic-induced cell death (Figure 4B–D). These data suggest that iron-dependent ferroptosis is not associated with corosolic acid-induced cell death.

Vitamin E was first explained in the 1920s as a dietary factor [33] and includes two groups, such as tocopherols and tocotrienols. α-Tocopherol acts as a hydrophobic antioxidant and possesses protective effects against free radical damage [34,35]. Moreover, α-tocopherol has been shown to exert protective roles for cancer, atherosclerosis, and neuronal damage [36,37,38,39]. A previous study reported that α-tocopherol protected against RSL3-induced lipid peroxidation, ROS production, and cell death in acute lymphoblastic leukemia cells [29]. Consistent with these results, we also detected α-tocopherol inhibited corosolic acid-induced generation of intracellular ROS, lipid peroxidation, and cell death (Figure 5B–D). However, lipid oxidation inhibitors (ferrostatin-1 and DFO) partially reduced corosolic acid-induced lipid oxidation, but not cell death. Stephane et al. reported that α-tocopherol has an inhibitory effect on several protein kinase inhibitor-mediated cytotoxic responses, which is independent of its antioxidant properties [40]. Therefore, further investigation is required to understand the exact mechanism of corosolic acid-induced cell death. In addition, it will be interesting study how α-tocopherol inhibits corosolic acid-cell death independently of its antioxidant function.

In conclusion, our results support that corosolic acid increases non-apoptotic cell death through induction of lipid ROS in human renal carcinoma cells.

## 4. Materials and Methods

### 4.1. Cell Cultures and Materials

American Type Culture Collection supplied all human cancer cells (renal carcinoma: Caki, ACHN, and A498, hepatocellular carcinoma: SK-hep1 and Huh7, breast carcinoma: MDA-MB-231) and the mouse kidney cells (TCMK-1) (Manassas, VA, USA). Primary culture of human mesangial cells (Cryo NHMC) were purchased from Clonetics (San Diego, CA, USA). Cells were grown in DMEM supplemented with 10% FBS and 100 µg/mL gentamycin. R&D system supplied z-VAD-fmk and TNF-α (Minneapolis, MN, USA), and Calbiochem supplied necrostatin-1, *N*-acetylcysteine (NAC) and trolox (San Diego, CA, USA). Santa Cruz Biotechnology supplied Anti-AIF antibody (Dallas, TX, USA), and Cell Signaling Technology supplied anti-PARP antibody (Beverly, MA, USA). Selleckchem supplied RLS3 (Houston, TX, USA), and BD Biosciences supplied anti-RIP1 antibody (San Jose, CA, USA). Sigma Chemical Co. supplied anti-actin antibody, Corosolic acid, cycloheximide (CHX), deferoxamine (DFO), ferrostatin-1, and α-tocopherol (St. Louis, MO, USA).

### 4.2. Cell Viability Assay

We used XTT assay to measure the cell viability (WelCount™ Cell Viability Assay Kit, WelGENE, Daegu, Korea). Briefly, after drug treatment, we added reagents to each well, and then incubated the plates. We detected the results using a multi-well plate reader (at 450 nm).

### 4.3. Lactate Dehydrogenase (LDH) Release Assay

For detection of cell death, we estimated the amount of released LDH into the cell culture medium using an LDH assay kit (Roche Molecular Biochemicals, Mannheim, Germany).

### 4.4. Asp–Glu–Val–Asp–ase (DEVDase) Activity Assay

To evaluate DEVDase activity, cell were treated with corosolic acid or TNF-α plus CHX, and 20 µg of cell lysates were incubated with reaction buffer as described in our previous studies [18]. We measured caspase activity at 405 nm absorbance using a spectrophotometer.

### 4.5. Western Blot Analysis

Whole cell lysates were obtained as described previously using modified RIPA [18,19,20]. Lysates were centrifuged at 13,000× *g* for 15 min at 4 °C, and the supernatant fractions were collected. Proteins were separated by SDS-PAGE and transferred to an Immobilon-P membrane. Specific proteins were detected using enhanced chemiluminescence.

### 4.6. Annexin V and 7-AAD Staining and Propidium Iodide (PI) Uptake

FITC-conjugated Annexin V and 7-aminoactinomycin D (7-AAD) (BD Pharmingen, San Jose, CA, USA) were used to estimate cell death mode. Cells were washed in cold PBS twice and resuspended in Annexin V-binding buffer (500 µL). We added 5 µL of Annexin V-FITC and 5 µL 7-AAD into the suspended cells, and then incubated for 15 min at room temperature in the dark. For PI uptake, cells in 100 µL of binding buffer were stained with 5 µg/mL propidium iodide and/or 10 µg/mL Hoechst33342 for 15 min at room temperature. After addition of 400 µL of binding buffer, the cell death population was detected by fluorescence-activated cell sorting (FACS) on a FACSCanto II (BD Biosciences, San Diego, CA, USA).

### 4.7. Small Interfering RNA (siRNA)

Santa Cruz Biotechnology supplied siRNA (RIP1 and AIF) and Bioneer supplied GFP (control) siRNA (Daejeon, Korea). We used Lipofectamine^®^ RNAiMAX Reagent (Invitrogen, Carlsbad, CA, USA) to transfect siRNA oligonucleotides.

### 4.8. Measurement of ROS

We used 2′,7′-dichlorodihydrofluorescein diacetate (H_2_DCFDA) to detect intracellular ROS. After treatment, cells were stained with the H_2_DCFDA for 10 min, and then washed with PBS twice. Fluorescence of cells in PBS was measured with a flow cytometer (BD Biosciences, San Jose, CA, USA) or fluorescence microscope (Carl Zeiss, Jena, Germany).

### 4.9. Detection of Lipid Peroxidation

After treatment, cells were stained with the 2.5 µM BODIPY-C11 fluorescent dye for 10 min (Invitrogen, Carlsbad, CA, USA). Then, cells were washed with PBS twice, trypsinized, and resuspended in PBS. Fluorescence was measured with a flow cytometer.

### 4.10. Statistical Analysis

The data were analyzed using a one-way ANOVA and post-hoc comparisons (Student–Newman–Keuls) using the Statistical Package for Social Sciences 22.0 software (SPSS Inc., Chicago, IL, USA). The *p* values < 0.05 were considered significant.

## Abbreviations

ROS	Reactive oxygen species
STAT	Signal transducer and activator of transcription
CHX	Cycloheximide
PARP	Poly (ADP-ribose) polymerase
7-AAD	7-Aminoactinomycin D
PI	Propidium iodide
RIP1	Receptor-interacting protein kinase1
AIF	Apoptosis-inducing factor
NAC	*N*-acetyl-l-cysteine
DFO	Deferoxamine

## References

[1] Devasagayam T.P., Tilak J.C., Boloor K.K., Sane K.S., Ghaskadbi S.S., Lele R.D. (2004). Free radicals and antioxidants in human health: Current status and future prospects. J. Assoc. Physicians India.

[2] Boonstra J., Post J.A. (2004). Molecular events associated with reactive oxygen species and cell cycle progression in mammalian cells. Gene.

[3] Toyokuni S., Okamoto K., Yodoi J., Hiai H. (1995). Persistent oxidative stress in cancer. FEBS Lett..

[4] Bielski B.H., Arudi R.L., Sutherland M.W. (1983). A study of the reactivity of HO2/O2- with unsaturated fatty acids. J. Biol. Chem..

[5] Gaschler M.M., Stockwell B.R. (2017). Lipid peroxidation in cell death. Biochem. Biophys. Res. Commun..

[6] Ayala A., Munoz M.F., Arguelles S. (2014). Lipid peroxidation: Production, metabolism, and signaling mechanisms of malondialdehyde and 4-hydroxy-2-nonenal. Oxid. Med. Cell. Longev..

[7] Barrera G. (2012). Oxidative stress and lipid peroxidation products in cancer progression and therapy. ISRN Oncol..

[8] Hou W., Li Y., Zhang Q., Wei X., Peng A., Chen L., Wei Y. (2009). Triterpene acids isolated from *Lagerstroemia speciosa* leaves as α-glucosidase inhibitors. Phytother. Res..

[9] Liby K.T., Yore M.M., Sporn M.B. (2007). Triterpenoids and rexinoids as multifunctional agents for the prevention and treatment of cancer. Nat. Rev. Cancer.

[10] Fukushima M., Matsuyama F., Ueda N., Egawa K., Takemoto J., Kajimoto Y., Yonaha N., Miura T., Kaneko T., Nishi Y. (2006). Effect of corosolic acid on postchallenge plasma glucose levels. Diabetes Res. Clin. Pract..

[11] Xu Y., Ge R., Du J., Xin H., Yi T., Sheng J., Wang Y., Ling C. (2009). Corosolic acid induces apoptosis through mitochondrial pathway and caspase activation in human cervix adenocarcinoma HeLa cells. Cancer Lett..

[12] Sung B., Kang Y.J., Kim D.H., Hwang S.Y., Lee Y., Kim M., Yoon J.H., Kim C.M., Chung H.Y., Kim N.D. (2014). Corosolic acid induces apoptotic cell death in HCT116 human colon cancer cells through a caspase-dependent pathway. Int. J. Mol. Med..

[13] Uto T., Sakamoto A., Tung N.H., Fujiki T., Kishihara K., Oiso S., Kariyazono H., Morinaga O., Shoyama Y. (2013). Anti-proliferative activities and apoptosis induction by triterpenes derived from *Eriobotrya japonica* in Human Leukemia Cell Lines. Int. J. Mol. Sci..

[14] Cai X., Zhang H., Tong D., Tan Z., Han D., Ji F., Hu W. (2011). Corosolic acid triggers mitochondria and caspase-dependent apoptotic cell death in osteosarcoma MG-63 cells. Phytother. Res..

[15] Nho K.J., Chun J.M., Kim H.K. (2013). Corosolic acid induces apoptotic cell death in human lung adenocarcinoma A549 cells in vitro. Food Chem. Toxicol..

[16] Lee M.S., Cha E.Y., Thuong P.T., Kim J.Y., Ahn M.S., Sul J.Y. (2010). Down-regulation of human epidermal growth factor receptor 2/neu oncogene by corosolic acid induces cell cycle arrest and apoptosis in NCI-N87 human gastric cancer cells. Biol. Pharm. Bull..

[17] Fujiwara Y., Komohara Y., Ikeda T., Takeya M. (2011). Corosolic acid inhibits glioblastoma cell proliferation by suppressing the activation of signal transducer and activator of transcription-3 and nuclear factor-κ B in tumor cells and tumor-associated macrophages. Cancer Sci..

[18] Jia Y., Yuan H., Shan S., Xu G., Yu J., Zhao C., Mou X. (2016). Corosolic acid inhibits the proliferation of osteosarcoma cells by inducing apoptosis. Oncol. Lett..

[19] Wang C.Y., Mayo M.W., Baldwin A.S. (1996). TNF- and cancer therapy-induced apoptosis: Potentiation by inhibition of NF-κB. Science.

[20] Darzynkiewicz Z., Juan G., Li X., Gorczyca W., Murakami T., Traganos F. (1997). Cytometry in cell necrobiology: Analysis of apoptosis and accidental cell death (necrosis). Cytometry.

[21] Zhang D., Lin J., Han J. (2010). Receptor-interacting protein (RIP) kinase family. Cell. Mol. Immunol..

[22] Delavallee L., Cabon L., Galan-Malo P., Lorenzo H.K., Susin S.A. (2011). AIF-mediated caspase-independent necroptosis: A new chance for targeted therapeutics. IUBMB Life.

[23] Cabon L., Galan-Malo P., Bouharrour A., Delavallee L., Brunelle-Navas M.N., Lorenzo H.K., Gross A., Susin S.A. (2012). BID regulates AIF-mediated caspase-independent necroptosis by promoting BAX activation. Cell Death Differ..

[24] Simon H.U., Haj-Yehia A., Levi-Schaffer F. (2000). Role of reactive oxygen species (ROS) in apoptosis induction. Apoptosis.

[25] Wang J., Yi J. (2008). Cancer cell killing via ROS: To increase or decrease, that is the question. Cancer Biol. Ther..

[26] Xie Y., Hou W., Song X., Yu Y., Huang J., Sun X., Kang R., Tang D. (2016). Ferroptosis: Process and function. Cell Death Differ..

[27] Dixon S.J., Lemberg K.M., Lamprecht M.R., Skouta R., Zaitsev E.M., Gleason C.E., Patel D.N., Bauer A.J., Cantley A.M., Yang W.S. (2012). Ferroptosis: An iron-dependent form of nonapoptotic cell death. Cell.

[28] Yang W.S., Stockwell B.R. (2008). Synthetic lethal screening identifies compounds activating iron-dependent, nonapoptotic cell death in oncogenic-RAS-harboring cancer cells. Chem. Biol..

[29] Probst L., Dachert J., Schenk B., Fulda S. (2017). Lipoxygenase inhibitors protect acute lymphoblastic leukemia cells from ferroptotic cell death. Biochem. Pharmacol..

[30] Carpenter K.L., Kirkpatrick P.J., Weissberg P.L., Challis I.R., Dennis I.F., Freeman M.A., Mitchinson M.J. (2003). Oral α-tocopherol supplementation inhibits lipid oxidation in established human atherosclerotic lesions. Free Radic. Res..

[31] Vandenabeele P., Galluzzi L., Vanden Berghe T., Kroemer G. (2010). Molecular mechanisms of necroptosis: An ordered cellular explosion. Nat. Rev. Mol. Cell Biol..

[32] Yu X., Deng Q., Bode A.M., Dong Z., Cao Y. (2013). The role of necroptosis, an alternative form of cell death, in cancer therapy. Expert Rev. Anticancer Ther..

[33] Evans H.M., Bishop K.S. (1922). On the existence of a hitherto unrecognized dietary factor essential for reproduction. Science.

[34] Ricciarelli R., Zingg J.M., Azzi A. (2001). Vitamin E: Protective role of a Janus molecule. FASEB J..

[35] Traber M.G., Atkinson J. (2007). Vitamin E, antioxidant and nothing more. Free Radic. Biol. Med..

[36] Mileva M., Bakalova R., Tancheva L., Galabov A., Ribarov S. (2002). Effect of vitamin E supplementation on lipid peroxidation in blood and lung of influenza virus infected mice. Comp. Immunol. Microbiol. Infect. Dis..

[37] Rao M.V., Sharma P.S. (2001). Protective effect of vitamin E against mercuric chloride reproductive toxicity in male mice. Reprod. Toxicol..

[38] Sigounas G., Anagnostou A., Steiner M. (1997). *dl*-α-tocopherol induces apoptosis in erythroleukemia, prostate, and breast cancer cells. Nutr. Cancer.

[39] Pratico D., Tangirala R.K., Rader D.J., Rokach J., FitzGerald G.A. (1998). Vitamin E suppresses isoprostane generation in vivo and reduces atherosclerosis in ApoE-deficient mice. Nat. Med..

[40] Pedeboscq S., Rey C., Petit M., Harpey C., De Giorgi F., Ichas F., Lartigue L. (2012). Non-antioxidant properties of α-tocopherol reduce the anticancer activity of several protein kinase inhibitors in vitro. PLoS ONE.

